# Modified penile reconstruction in classic bladder exstrophy: Can complete corporal covering of the urethral closure be achieved using incomplete disassembly technique?

**DOI:** 10.1590/S1677-5538.IBJU.2024.0194

**Published:** 2024-06-15

**Authors:** Vasily V. Nikolaev, Nikita V. Demin

**Affiliations:** 1 Pirogov Russian National Research Medical University Moscow Russia Pirogov Russian National Research Medical University, Moscow, Russia; 2 Clinical and Research Institute of Emergency Pediatric Surgery and Trauma Moscow Russia Clinical and Research Institute of Emergency Pediatric Surgery and Trauma, Moscow, Russia

**Keywords:** Epispadias, Bladder Exstrophy, Penis

## Abstract

**Purpose:**

To answer the question of whether it is possible to achieve complete corporal covering of the urethral closure using incomplete penile disassembly in classic bladder exstrophy. We hypothesize that mobilization of the corpora under Buck's fascia, their dorsal translocation through the incisions in Buck's fascia and suturing corporal convex sides above the urethra would allow extend corporal covering of the urethra, reducing the risk of urethra-cutaneous fistula formation.

**Materials and Methods:**

A prospective follow-up on all boys who underwent the modified Cantwell-Ransley primary penile reconstruction was conducted. Inclusion criteria comprised bladder exstrophy closure in our institution, ensuring a postoperative follow-up period of no less than 24 months. The key innovation of the technique lies in a deep dissection of the dependent corpora under Buck's fascia, followed by their dorsal relocation through extended dorsal incisions in Buck's fascia, and limited external corporal rotation 90 degrees only at the base of the penis.

**Results:**

Between November 2019 and March 2022, 18 boys aged 11 to 35 months met the inclusion criteria and underwent the modified penile reconstruction. Surgical procedures and postoperative period did not include any major complications. Total corporal covering of the urethral sutures was achieved in 15 of 18 patients. No urethra-cutaneous fistulas were observed within 2 years of follow-up. All individuals demonstrated spontaneous erections, and the absence of dorsal curvature was documented.

**Conclusion:**

The modified technique of incomplete penile disassembly applied in a homogenous group of patients with classic bladder exstrophy allows penile shaft elongation, improved aesthetic outcomes, preserved erections, and eliminates dorsal curvature. The technique demonstrated feasibility and reliability while maintaining positive effects on tissue circulation. The absence of urethra-cutaneous fistulae is attributed to the complete corporal covering of the urethral sutures and supports the initial hypothesis.

## INTRODUCTION

Penile reconstruction in individuals with classic bladder exstrophy (CBE) represents a formidable challenge. The implementation of penile disassembly techniques, involving the ventral relocation of the urethra during the latter part of the 20th century has notably enhanced aesthetic results and diminished complication rates. Despite these advancements, issues such as diminished penile shaft size and recurring dorsal curvature (1, 2) persist as prevalent concerns following epispadias repair, prompting the need for ongoing technical refinements (3).

Currently, Cantwell-Ransley repair modifications or incomplete penile disassembly (IPD) techniques are extensively employed (4-7). In IPD techniques, the cavernous bodies (CB) remain dependent — affixed to the glans penis on one side and to the ischio-pubic rami on the other. The urethral plate is not entirely disassembled from the glans. A distinctive characteristic of these approaches involves the internal rotation of the corpora, generally without the mobilization of neurovascular bundles (NVB). The corpora are approximated at the midline above the urethra by their short concave sides, housing inextensible intrinsic chords. This configuration results straightening and also shortening of the penile shaft (PS) and a deficiency in corporal covering of proximal urethral sutures, a factor believed to be linked to the development of urethra-cutaneous fistula (8-10). The Manchester group (11) recently reported external rotation of the corpora without Buck's fascia elevating in incomplete penile disassembly, which also did not lead to penile shaft lengthening and complete coverage of the urethral sutures due to lateral tension of the corpora.

We hypothesize that by mobilizing the corpora from Buck's fascia, relocating them over the urethra through the extended dorsal incisions in Buck's fascia, and suturing together the longer ventral corporal surfaces, it would be possible to lengthen and straighten penile shaft and extend the corporal covering of the urethra, reducing the risk of urethra-cutaneous fistula formation. To implement this idea, several modifications were introduced into the incomplete penile disassembly technique and are presented in this report along with mid-term results.

## MATERIALS AND METHODS

The study protocol was approved by the Research Ethics Committee (approval number REB# 1000054434) 19 September 2019.

We conducted a prospective follow-up on all boys with classic bladder exstrophy who underwent primary penile reconstruction by employing a modified technique, ensuring a postoperative follow-up period > 24 months. Inclusion criteria comprised patients with CBE who underwent closure in our institution. Patients presented with very short urethral plates - less than 15 mm between the verumontanum and the tip of the glans were excluded. The detailed technique for exstrophy closure has been previously elucidated (12).

As part of the urinary bladder closure, the Johnston maneuver was executed, involving the separation of the anterior ⅔ of the CB from the ischiopubic rami, along with the periosteum, in preparation for subsequent epispadias repair (ER) (13). Additionally, pubic bones approximation preventing deepening of the penis was performed in conjunction with the described exstrophy closure procedure.

The pre-surgical protocol included the application of testosterone 2–3 times daily for a duration of 3 weeks. For cases of total urinary incontinence, two testosterone enanthate injections (2 mg/kg) were administered five and two weeks prior to the surgery.

### Surgical technique

The dissection of the skin was carried out along the borders of the urethral plate, encircling the neo-meatus, and extending along the corona. Lateral incisions of the skin, reaching down to the scrotum, were made to alleviate tissue tension and facilitate unimpeded access to the attachments of CB. We consider the reconstruction of the penile shaft as a simultaneous achievement of several objectives: maximizing the elongation of the penile shaft and achieving a cylindrical shape; transposing the corpora over the urethra; correcting any dorsal curvature; ensuring coverage of the urethral sutures with the corpora; preserving penile circulation.

### Corporal dissection

Corporal mobilization was conducted beneath BF through its longitudinal ventral incisions, extending from the glans corona down to the ischiocavernosus muscle (14). To safeguard the neurovascular bundles and the spongious body, they remained attached to BF, ensuring their protection and facilitating the overall technique. The cavernous bodies were deliberately left dependent, retaining their connection to the tip of the glans penis and the ischiopubic rami. In this way, distally the corpora are almost completely released, leaving only the apexes of the corpora connected to the glans (4, 5).

**Figure 1:**
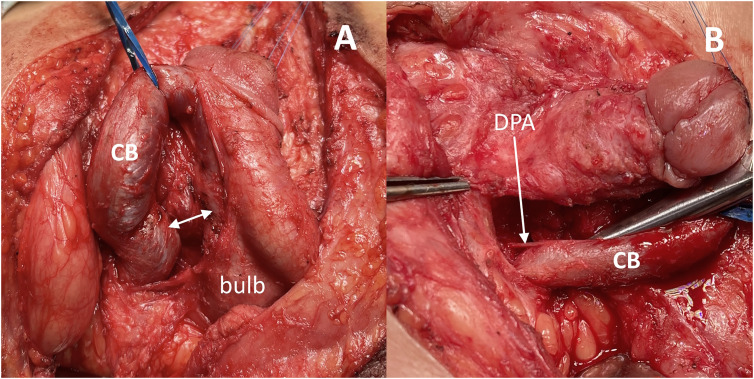
Cavernous body dissection.

Similarly, the urethral plate was not completely separated from the glans. An important step involved dissecting the corpora from the posterior part of the bulb, where a firm attachment restricted the mobility of CB. This medial corporal attachment was sharply dissected without significant bleeding (Figure-1A). Typically, at this level, the deep artery of the penis dorsally or dorsal-laterally enters the CB (Figure-1B), necessitating preservation due to its vital role in supplying the corpora (15).

A) Cavernous body is dissected from the urethral bulb (bulb). White arrow indicates the gap between the bulb and the right CB. B) Deep penile artery (DPA) dorsally enters the cavernous body – white arrow.

### Cavernous body relocation and urethroplasty.

**Figure 2:**
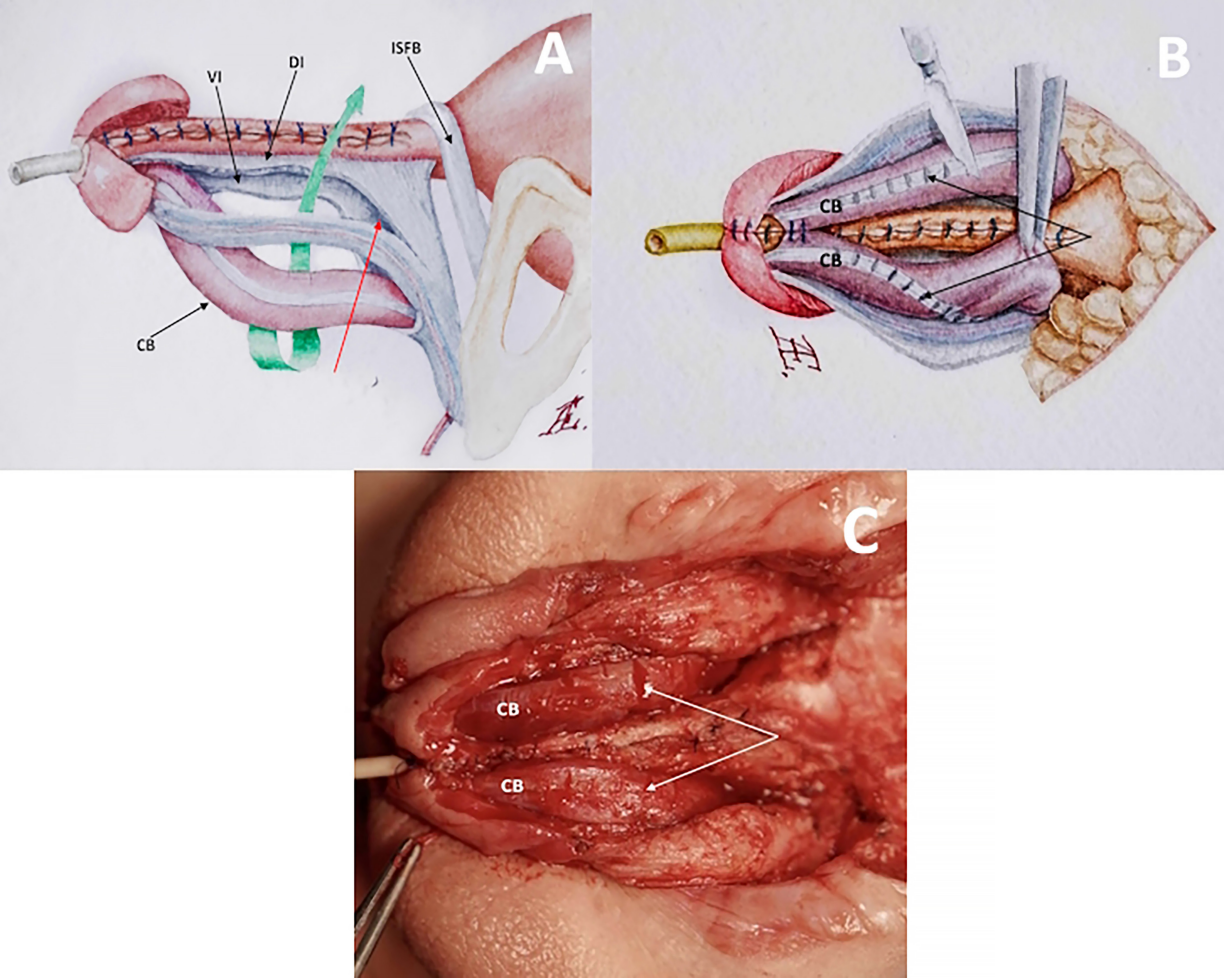
Cavernous body relocation.

Dorsal incisions were meticulously crafted between the edge of the urethral plate and the NCB, extending from the tip of the glans to the prostatic part of the urethra, with a dorsal cut of BF to the perineum (Figure-2A). It is crucial to note that the BF incision continues more proximally than its junction with the urethral plate, a significant factor in achieving maximal penile shaft length and complete corporal covering of the urethral closure. Tubularization of the urethral plate was executed using a continuous extramucosal 6/0 suture over a #8 Ch pigtail catheter. When the penis is deflected caudally, CB forms mobile loops, freely relocating dorsally through the extended incisions of BF. To relieve intrinsic chordee, transverse incisions were made across it (16). Subsequently, the convex sides of the corpora at the base of the penis were directed toward each other over the urethra (Figures 2B and C). In the described technique, rotation of the corpora at the base of the penis did not lead to significant torsion in the middle of the shaft or crura of the penis and deterioration of blood supply through the cavernous artery, due to extended proximal and distal corporal mobilization and a relatively small angle of rotation.

A) Dependent CB is prepared to be relocated dorsally along the green arrow. Dorsal incision of Buck's fascia between the neurovascular bundle and the urethral plate stretches from the glans to the pelvic floor level (red arrow) extending more proximally to the junction with the urethral plate. The inter-symphisal fibrous band (ISFB) is located at the level of the pelvic floor. It is apparent that the neurovascular bundle remains attached to Buck's fascia. The urethra is tubularized. DI - dorsal incision, VI - ventral incision. B) Counter torsion of the corpora. After the attenuations on the chordee are made, the convex ventral sides of the corpora are turned medially (as shown with the forceps) at the base of the penis, while no torsion is noted at the apical portion of corpora. The corpora are sutured in the middle of the glans. C) Cavernous bodies were moved dorsally through the incisions in Buck's fascia. Noteworthy is the absence of mid-corporal torsion. Releasing transverse incisions (attenuations) are made on the chordee (arrows).

### Penile shaft assembly

To effectively address the curvature of PS and establish a cylindrical penile shape, a series of Gittes tests are conducted during the assembly process. The initial suture is strategically placed between the convex sides of the corpora over the urethra, positioned more proximally than the initiation point of urethral closure. This suture serves as the proximal edge of the penile shaft.

**Figure 3:**
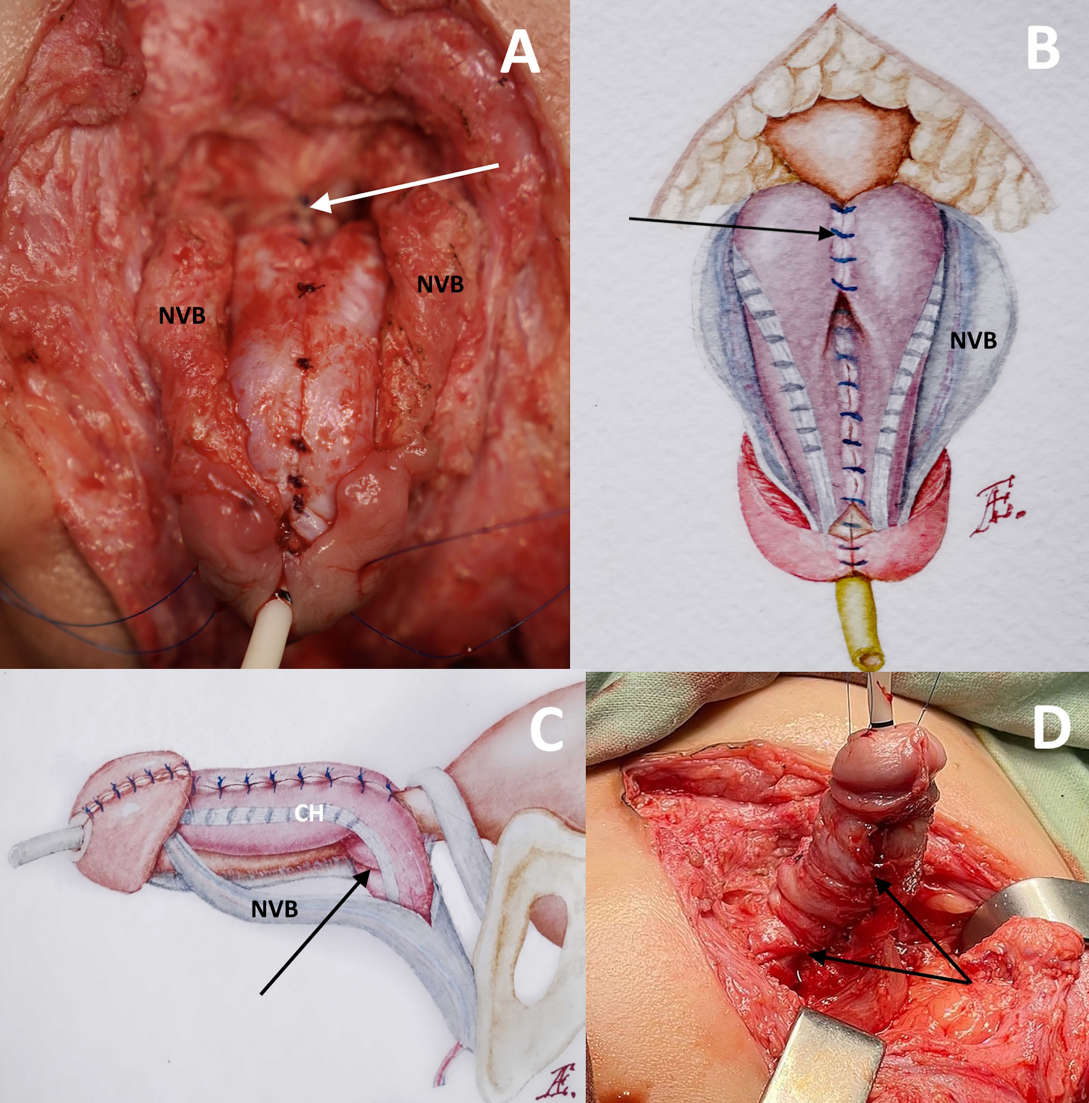
Penile shaft assembly.

**Figure 4:**
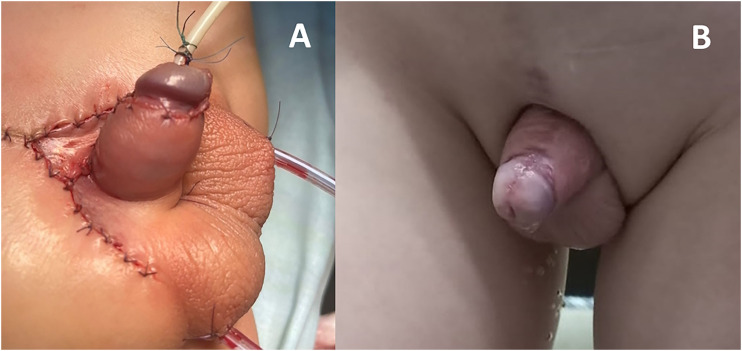
Results of penile reconstruction at the end of the procedure (A) and at mid-term follow-up (B).

The second suture is delicately placed over the urethra between the corpora in the middle of the glans, with only slight internal corporal rotation in this place ensuring a tension-free application. Notably, the apical segments of the corpora are deliberately left unsutured to avoid potential circulatory complications in the glans and the distal section of the urethra. The closure of the corpora along the line connecting these two sutures results in the formation of the PS. Therefore, only corporal segments at the base of the penile shaft were rotated externally 90 degrees ensuring covering the urethral sutures below the prostate (Figure-3A). Dorsal intercorporeal sutures are duplicated to reinforce the base of the penis. Subsequently, a conventional glanduloplasty is performed (Figures 3B and C). The restoration of the fascial sheath of the PS is executed without tension to prevent tethering (Figure-3D). Specifically, only the superficial fascia is closed along the midline dorsally, with no approximation of NVB in the midline. For skin coverage, parascrotal skin flaps and penile skin are employed (Figure-4A).

A and B) The CB are joined along the midline. The base of the penis is reinforced by the security stitches to prevent dehiscence during erections. White arrow - narrowing suture is placed on the prostatic urethra. Black arrow - second row of stitches at the base of the penis. All the sutures of the urethral closure are covered with the CB. C) Side view after penile shaft reconstruction. As a result of relocation and the approximation of the convex sides of the corpora, the normal Г-shape of the CB is restored. The chordae (Ch) at the base of the penis are located laterally - the curvature is corrected. A cylindrical shape of the penis has been achieved. Black arrow - subtle torsion of the penile crus. D) Sutures attaching Buck's fascia to the albuginea are marked with black arrows. Restored fascial sheath of the penile shaft.

Characteristic features of the appearance of the penis following the implementation of the described technique: visible shaft of the penis, which has a cylindrical shape; lack of curvature; orthotopic position of the meatus; normal angle of deflection of the penis during spontaneous erection.

### Postoperative assessment

All patients underwent follow-up assessments for 24 months post-surgery (Figure-4B). The evaluation encompassed the visual appraisal of the penis, its configuration during spontaneous erections, and urination, conducted both in an outpatient setting and through the analysis of photographs and video recordings of urination. Comprehensive examinations of the children occurred at the outpatient facility at intervals of 1, 3, 6, 12, 24 months following surgical intervention.

## RESULTS

Between November 2019 and March 2022, penile reconstruction was conducted on 38 patients diagnosed with CBE. Out of this cohort, 18 boys aged 11 to 35 months satisfied the inclusion criteria and underwent the modified primary penile reconstruction and epispadias repair as part of staged repair of bladder exstrophy.

Surgical procedures and postoperative phases were notably devoid of any major complications. The developed surgical technique was performed in a similar manner in this series without osteotomy or pubic bones approximation. Total corporal covering of the urethral sutures was achieved in 15 of 18 patients. In three cases, challenging micturition following catheter removal necessitated the reinsertion of the catheter for an additional two weeks, coupled with a course of antibiotics. Local skin wound dehiscence (Clavien-Dindo - I) without urinary leakage was noted in 5 cases.

Postoperatively, aesthetic outcome was evaluated by two ambulatory urologists as good in 14 and satisfactory in 4 the patients with vertical meatus at the tip of the conical glans, a straight penis, and pliable penile skin (Figure-4). Unobstructed urination was further noted in all boys. Notably, no urethra-cutaneous fistulas were observed within this patient cohort. Twelve boys showed an increase in the volumes of morning micturition, and the duration of dry intervals at rest.

All individuals demonstrated spontaneous erections, and the absence of dorsal curvature was consistently noted and documented through video and photographic evidence during the 24 months post-discharge period (Figures 4A and B). Each patient exhibited a PS that maintained a straight configuration, deviating at an angle of 90±20 degrees from the frontal plane during spontaneous erections. At the midterm follow-up, all cases demonstrated the attainment of a correct cylindrical shape and displayed proper aesthetic features of the penis.

## DISCUSSION

Penile reconstruction and ER in patients with CBE significantly advanced in the late 20th century. Such important achievements in reconstructive techniques for epispadias repair include penile disassembly (4, 10) and method for correcting the dorsal curvature of the penis by rotating the corpora, which was invented by Stephen Koff (17). The author used outward rotation of corpora without ventralization of the urethra in Young's epispadias repair. Subsequently, external or internal rotation of the corpora was adopted in most modifications of penile disassembly (4, 5, 18).

Notably, Cantwell-Ransley modifications of incomplete disassembly in epispadias repair, widely employed by experts, yield acceptable results (19). These techniques carry a lower risk of ischemic disorders and avoid hypospadias, distinguishing them from more radical approaches such as the Kelly soft tissue mobilization or the Mitchell Complete Penile Disassembly technique (10, 20-22).

Distinctive features of IPD techniques encompass preserved connection of the glans and the corpora to the urethral plate, coupled with the inward rotation of the corpora over the urethra to eliminate dorsal curvature. While corporal inward rotation successfully corrects curvature in many cases without the mobilization of the NVB, most of modifications (4, 5) involve suturing the short, concave sides of the corpora, which contain the least elastic parts of the albuginea known as intrinsic chordae. This inevitably results in penis shortening. Consequently, the sutures of the tubularized urethra at the base of the penis remain uncovered by the corpora, posing a well-established risk for the dehiscence or urethra-cutaneous fistula formation. (8, 24, 25). Their treatment is difficult and sometimes requires 3 to 6 operations (26). Furthermore, these modifications to the Cantwell-Ransley technique fail to produce a cylindrical penis shape, as the convex sides of the corpora end up on the exterior following inward rotation, potentially causing a waist-like deformity at the base of the penis (23).

Our hypothesis posited that by suturing the longer, convex sides of the dependent corpora over the urethra in incomplete penile disassembly technique such as modified Cantwell-Ransley (4, 5), it would be possible to achieve penile shaft lengthening, covering of urethral sutures, and correct dorsal curvature. This conceptualization was actualized in the described technique, which was uniformly applied in a prospective study involving a homogeneous series of patients undergoing primary ER within 6-11 months after exstrophy closure.

The proposed technique effectively attains the aforementioned goals of penile shaft reconstruction through a series of consecutive steps. The key innovation of this technique lies in a more proximal subfascial corporal dissection for releasing and lengthening of the dependent corpora, followed by their dorsal relocation through extended dorsal incisions in Buck's fascia and limited 90-degree external rotation of the short corporal segments at the base of the penis to avoid a significant mid-corporal torsion and subsequent circulatory problems. In the existing literature, we did not find a description of a technique combining the reconstruction of the penile shaft in a similar manner.

With the CB in a dependent position and the urethra, along with the spongiosum, attached to the glans, achieving free corporal relocation necessitates a deeper mobilization of the corpora from Buck's fascia. A crucial aspect of this dissection involves the complete detachment of the medial surface of the corpora from the posterior aspect of the bulb, entailing the transection of the robust attachment between them. We didn't meet descriptions of this specific anatomical structure or features of the dissection of the corporal-bulbar connection at this particular location. Corporal mobilization to the tip of the glans is also important for free CB relocation and rotation. Achieving penile shaft elongation and the corporal covering above the urethral sutures involves pulling the mobilized CB dorsally through extended dorsal incisions on Buck's fascia. Correction of dorsal curvature and the formation of a cylindrical penile shaft are accomplished by equalizing the length of all corporal sides. This is achieved by orienting the corporal convex sides at the base of the penis toward each other and closing them above the urethra. The chordee which was initially located dorsally, as a result of torsion of the penile crura 90 degrees outward, is moved at the base of the penis to the lateral side, where it ceases to have a bending effect. In this manner, the penis can be fully straightened, the corpora assume their normal Г-shape, and the bending moment of force curving the corpora dorsally approaches zero. We found that penile curvature correction by corporal rotation is somewhat more complex than described in the literature, since rotation of any corporal segment causes torsion between the fixed and rotated portions. The shorter the twisted parts and the greater the angle of rotation of the corpora, the greater the deformity and the risk of partial or complete obstruction of the cavernous artery.

In the described technique short rotated segments and extended mobilization of the corpora lengthen twisted areas, reducing local deformation, which apparently has a positive impact on blood supply. Moreover, the corpora mostly cover the urethral suture, extending from the neo-meatus to the center of the glans, thereby minimizing the risk of urethra-cutaneous fistula formation. Our prior experiences have indicated that the approximation of the distal segments of the corpora is not advisable, as it may compromise apical blood supply and lead to glans ischemia. We did not observe any disruption of corporal blood supply after its dissection from the bulb. Obviously, this technical point is delicate, like the technique of dissection of the cavernous bodies in general and requires experience in the penile disassembly techniques.

The penile shaft assembly technique is often overlooked in the literature, despite its pivotal role in determining the final shape and position of the penis. Notably, pronounced tethering can arise when NVB are approximated at the midline, a practice recommended by most authors conducting incomplete or complete penile disassembly. Therefore, our preference is to close dorsally only the superficial fascia while attaching the edges of Buck's fascia to the ventrolateral surface of the albuginea without tension.

The assessment of aesthetic and functional results involved both physical examinations and the review of photographic and video materials captured at rest and during spontaneous erections, provided by the parents. This comprehensive approach aimed to obtain tangible evidence of dorsal curvature correction and the shape of the penis during an erection in individuals with exstrophy. The inclusion of real-life evidence contributed to the verification of the outcomes achieved by the proposed evaluation methodology, making it viable for further evaluation and adoption.

Limitations of this study include a relatively small cohort of patients who underwent this procedure, specifically penile reconstruction as part of staged exstrophy repair, and the absence of a control group. The technique should be used by the surgeons experienced in penile disassembly techniques with caution to avoid circulatory disorders. Obviously, long-term results of the technique after puberty are required, but the mid-term aesthetic and functional results obtained are encouraging.

## CONCLUSIONS

The presented modified Cantwell-Ransley technique of incomplete penile disassembly with reconstruction was applied in a homogeneous group of patients with classic bladder exstrophy. The key innovation of the technique lies in the complete corporal detachment from the bulb followed by their dorsal relocation through extended dorsal incisions in Buck's fascia and a limited 90-degree external local corporal rotation at the base of the penis. The technique demonstrated feasibility and reliability while maintaining positive effects on tissue circulation, allowed penile shaft elongation, improved aesthetic outcomes, preserved erections, and eliminated dorsal curvature. The absence of urethra-cutaneous fistulae is attributed to the complete corporal covering of the urethral sutures which supports the initial hypothesis.

## ABBREVIATIONS

ER: = Epispadias repair
CBE: = Classic bladder exstrophy
IPD: = Incomplete penile disassembly
CB: = Cavernous bodies
NVB: = Neurovascular bundles
PS: = Penile shaft
BF: = Buck's fascia
